# Untargeted Metabolomics Reveals Predominant Alterations in Lipid Metabolism Following Light Exposure in Broccoli Sprouts

**DOI:** 10.3390/ijms160613678

**Published:** 2015-06-15

**Authors:** Mariateresa Maldini, Fausta Natella, Simona Baima, Giorgio Morelli, Cristina Scaccini, James Langridge, Giuseppe Astarita

**Affiliations:** 1 Food and Nutrition Research Centre, Consiglio per la ricerca in agricoltura e l’analisi dell’economia agraria (CRA), 00184 Roma, Italy; E-Mails: maldiniluce@gmail.com (M.M.); fausta.natella@entecra.it (F.N.); simona.baima@entecra.it (S.B.); giorgio.morelli@entecra.it (G.M.); cristina.scaccini@entecra.it (C.S.); 2 Department of Chemistry and Pharmacy, University of Sassari, 07100 Sassari, Italy; 3 Waters Corporation, Health Sciences, Milford, MA 01757, USA; E-Mail: James_langridge@waters.com; 4 Department of Biochemistry and Molecular & Cellular Biology, Georgetown University, Washington, DC 20057, USA

**Keywords:** metabolomics, lipidomics, lipids, ion mobility, nutrition, sterol lipids, oxylipins

## Abstract

The consumption of vegetables belonging to the family *Brassicaceae* (e.g., broccoli and cauliflower) is linked to a reduced incidence of cancer and cardiovascular diseases. The molecular composition of such plants is strongly affected by growing conditions. Here we developed an unbiased metabolomics approach to investigate the effect of light and dark exposure on the metabolome of broccoli sprouts and we applied such an approach to provide a bird’s-eye view of the overall metabolic response after light exposure. Broccoli seeds were germinated and grown hydroponically for five days in total darkness or with a light/dark photoperiod (16 h light/8 h dark cycle). We used an ultra-performance liquid-chromatography system coupled to an ion-mobility, time-of-flight mass spectrometer to profile the large array of metabolites present in the sprouts. Differences at the metabolite level between groups were analyzed using multivariate statistical analyses, including principal component analysis and correlation analysis. Altered metabolites were identified by searching publicly available and in-house databases. Metabolite pathway analyses were used to support the identification of subtle but significant changes among groups of related metabolites that may have gone unnoticed with conventional approaches. Besides the chlorophyll pathway, light exposure activated the biosynthesis and metabolism of sterol lipids, prenol lipids, and polyunsaturated lipids, which are essential for the photosynthetic machinery. Our results also revealed that light exposure increased the levels of polyketides, including flavonoids, and oxylipins, which play essential roles in the plant’s developmental processes and defense mechanism against herbivores. This study highlights the significant contribution of light exposure to the ultimate metabolic phenotype, which might affect the cellular physiology and nutritional value of broccoli sprouts. Furthermore, this study highlights the potential of an unbiased omics approach for the comprehensive study of the metabolism.

## 1. Introduction

The *Brassicaceae*, a family of widely consumed plants, includes broccoli, cabbage, kale, Brussels sprouts, and many other vegetables. The known healthful effects of ingesting these vegetables include a lower risk of developing cancer and cardiovascular diseases [1,2,3,4]. Yet the extent to which the effects of various growth conditions—particularly light exposure—affect the vegetables’ metabolism, and hence their nutritional value, remains incompletely characterized.

**Figure 1:**
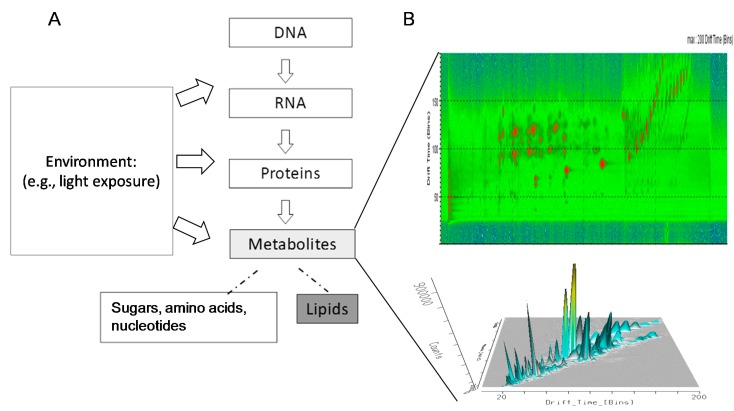
(**A**) Metabolomics aims to screen all the metabolites present in biological samples. Metabolites can derive from both the generic imprint and from the environment (e.g., light exposure). Metabolites are counted in the order of thousands and have a wide range of chemical complexity and concentration. The profiling of the entire set of metabolites—the metabolome—defines the molecular phenotype of the biological system; (**B**) Untargeted metabolomics were conducted using Ultra-Performance Liquid-Chromatography (UPLC) coupled with an ion mobility-enabled QTof MS. After UPLC separation (**top** panel), metabolites were further separated in another dimension using ion-mobility cell before MS detection (**center** and **bottom** panels). The combination of UPLC and ion mobility increased peak capacity and specificity in the quantification and identification process [19,20,21,22].

Young broccoli plants are especially enriched in antioxidant and chemoprotective metabolites, with levels several times greater than those of mature plants [5]. The molecular composition of broccoli sprouts reflects both genetic and environmental components. For that reason, comprehensive metabolite profiles can more completely describe the vegetables’ ultimate nutritional value than can genomics approaches (Figure 1A). Metabolomics is a modern analytical approach that uses state-of-the-art instrumentation, such as mass spectrometry (MS), to characterize the molecular composition of biological samples [6]. To date, metabolomics investigations of broccoli sprouts have mainly focused on “targeted metabolomics” approaches, thus focusing on analyzing selected molecular classes, including glucosinolates, isothiocyanates and anthocyanins [1,7,8,9,10,11,12,13,14].

A complementary approach, “untargeted metabolomics”, aims to screen the entire metabolite content of biological samples. Such an unbiased approach can be used for characterizing the molecular phenotype of individual samples or for comparing profiles of metabolites among different sample groups. Recent technological advances in the field of MS allow us to perform both qualitative and quantitative analysis of thousands of metabolites in a single analysis, providing novel opportunities to investigate the molecular response of broccoli sprouts and their nutritional value to environmental stimuli [6,15,16,17,18].

When germinated in the dark, in an attempt to reach a source of light, the sprouts undergo a developmental program called skotomorphogenesis characterized by great cell expansion driven by water uptake and consumption of the metabolic reserve accumulated into the seed. Therefore dark grown sprouts can be considered to have a minimal metabolic complexity. On the contrary, light exposure during germination induces the photomorphogenic program leading to the establishment of autotrophy. Due to the conversion of light energy into chemical energy and to the oxidative stress associated to the photosynthesis, light grown sprouts are characterized by a high metabolic activity.

In this study, we pursued an untargeted metabolomics approach to investigate the molecular changes occurring in the complete set of metabolites—the “metabolome”—of broccoli sprouts grown under conditions of light or dark. By comparing, in an unbiased fashion, the molecular information of such extreme growth conditions, we highlighted major biochemical pathways affected by light exposure, a circumstance that might ultimately affect the cellular physiology and nutritional value of broccoli sprouts.

## 2. Results and Discussion

To identify the major molecular alterations occurring in broccoli sprouts grown under conditions of a light/dark photoperiod, compared with conditions of complete darkness, we developed an untargeted metabolomics approach.

**Figure 2:**
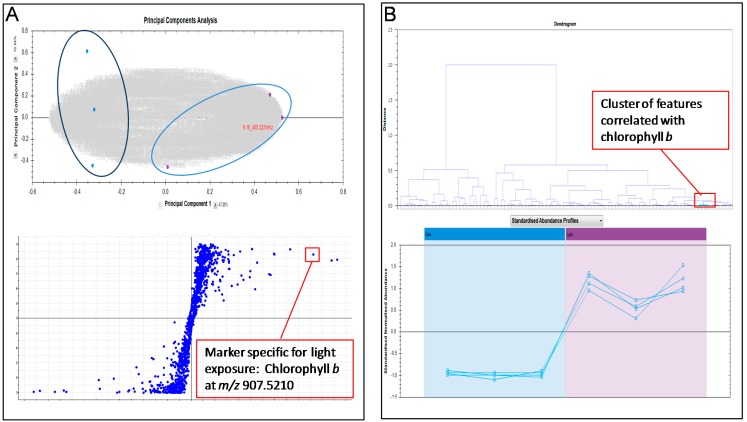
(**A**) Multivariate statistical analysis of the UPLC/HDMS^E^ runs allowed separating samples into clusters using PCA. The metabolites that contributed most to the variance among groups were isolated using least-squares discriminant analysis (PLS-DA; bottom left); (**B**) Correlation analyses helped to identify similar patterns of alterations among metabolites. A representative example is showed for the metabolite with *m*/*z* 907.5210, which is increased in the light exposed samples and was then identified as chlorophyll *b*.

To maximize the separation of the wide range of metabolites present in the broccoli sprouts, we used a combination of Ultra-Performance Liquid-Chromatography (UPLC) and ion-mobility separations (Figure 1B) [19,23,24]. The analysis provided a multidimensional metabolite fingerprint, which represented a “snapshot” of the metabolite inventory for each sample analyzed (Figure 1B). Differences at the metabolite level between groups were analyzed using multivariate statistical tools, including principal component analysis (PCA) and correlation analyses (Figure 2). The metabolites that contributed most to the variance between groups were isolated using PLS-DA and ANOVA (Figure 2).

**Figure 3:**
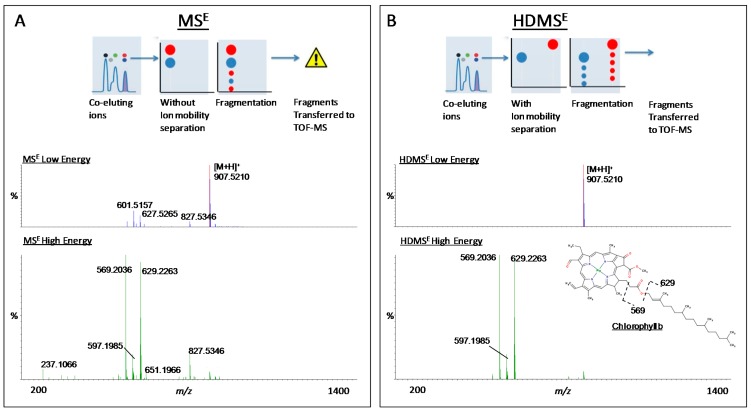
Representative MS^E^ (**A**) and HDMS^E^ (**B**) acquisitions of both precursors and fragment spectra information along one single chromatographic run. Applying high collision energy in the transfer collision cell, precursor molecules can be broken down into constituent parts (product ions), to deduce the original structure (bottom panel). To help identify complex mixtures of metabolites, the identification of the chlorophyll *b* structure was based on the observation of characteristic fragments generated with high energy after ion-mobility separation using (HDMS^E^). The inclusion of an ion-mobility separation of co-eluting precursor metabolites by HDMS^E^ produced cleaner product ion spectra compared to MS^E^, which facilitated the identification of chlorophyll *b* by searching against databases and previously published results [27].

Metabolite identification is a critical step for converting data into meaningful, biological results. In a typical MS-based metabolomics experiment, features of interest are usually searched against databases that list physicochemical properties descriptive of each metabolite (e.g., accurate mass). Initial searches, performed using publicly available and in-house databases, led to more than 700 tentative identifications of metabolites that accumulated in the broccoli sprouts exposed to light (Table S1). To aid in the identification and structural elucidation of metabolites, data-independent acquisition was coupled with ion-mobility separation in a particular mode of operation: high definition MS^E^ (HDMS^E^) [19,20,21,22,23,24,25]. Because many molecules in complex matrices co-elute, the incorporation of ion mobility allowed the separation of ions before fragmentation, producing cleaner, tandem-MS, product-ion spectra that facilitated metabolite identification (Figure 3 and Figure S1) [19,20,21,22,25,26].

**Figure 4:**
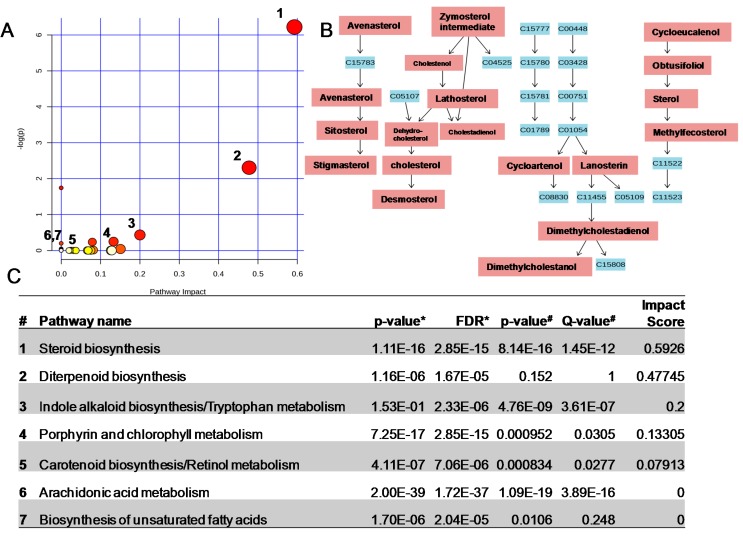
(**A**) Summary of pathway analysis offers a metabolomics view, which displays all matched pathways as circles. The color and size of each circle is based on the *p* value and pathway impact value, respectively. Please refer to Table S2 for numerical details; (**B**) Representation of the steroid biosynthetic pathway. In red, the metabolites that increased in broccoli sprouts grown under conditions of continuous light, compared with the metabolites in sprouts grown under conditions of continuous dark. In blue, the KEGGS numbers are reported for each metabolite in the same pathway that do not appear to be altered; (**C**) Summary of the major metabolic pathways altered in broccoli sprouts grown under conditions of continuous light, compared with the metabolites in sprouts grown under conditions of continuous dark. False Discovery Rate (FDR*) and *p*-value * from MPINet [29]; *p*-value ^#^ and *Q*-value ^#^ from IMPaLA [30]; impact scores for the topological analysis using relative betweeness centrality from MetPA [28].

An obvious limitation of this approach is that it is impractical to verify the identification for each potential metabolites, including isomers, isobars and other unlikely plant metabolites (Table S1). Thus, tentative identifications were compared with 87 pathways that appear in the Kyoto Encyclopedia of Genes and Genomes (KEGG) pathway library of *Arabidopsis thaliana* (thale cress), a member of the same family, *Brassicaceae*, as the broccoli sprouts (Table S2 and Figure 4) [28]. By testing whether a set of metabolites is enriched in a particular pathway, compared with random hits, this approach allowed to support metabolite identification. Because metabolite changes are interconnected and occur in a coordinated fashion in biology, finding multiple metabolite hits within a particular biochemical pathway increases the probability that the identification is correct. The use of additional over-representation analysis tools originally developed for mammalian metabolism, including MPINet and IMPaLA, further supported the validity of our initial observations (Tables S3 and S4) [29,30]. Such complementary metabolomic pathway analyses helped us to identify subtle but significant changes among groups of related metabolites that may have gone unnoticed with conventional approaches (Figure 4).

Besides the predictable alterations in chlorophyll biosynthesis (Figure 4), we observed an increase in the levels of phytosterols, prenol lipids as well as carotenoids and polyunsaturated fatty acids-containing lipids in broccoli sprouts grown under conditions of light/dark cycle, compared with conditions of continuous dark (Figure 4 and Tables S1–S4). Sterol lipids are known to play key roles in the growth and development of plants and to contribute to controlling the expression of genes linked to photosynthesis [31,32]. The increase in carotenoid species is known to help plants absorb light energy and to protect chlorophyll against photooxidative stress [33,34]. The increase in the desaturation of fatty acids has been proposed as an adaptive response to shifts in light intensity [35,36]. The remodeling of membrane fluidity might, indeed, affect lipid-protein interactions, including the self-assembly of active chlorophyll-protein complexes for photosynthetic apparatus [35,36]. Thus the concomitant activation of the steroid, chlorophyll, carotenoid, and polyunsaturated fatty acid (PUFA) pathways by light exposure might work synergistically for the engagement of the photosynthetic machinery. Notably, carotenoids and PUFA are both essential to human health, and they are absorbed through diet [37,38,39,40]. Consumption of phytosterols affects the endogenous sterol lipid metabolism and has been linked to decreased cardiovascular diseases and cancer [41,42,43]. 

We also found that light exposure increased the levels of various polar metabolites belonging to the category of polyketides, including flavonoids (Table S1). These molecules, known to possess strong antioxidant properties, have been associated with health-promoting benefits [44,45,46]. Our results are in agreement with previous observations showing that the levels of polyketides are affected by environmental conditions including temperature and light conditions [5,7,11,47,48,49,50,51,52,53,54,55,56,57,58]. These observed changes in phytochemical composition following light exposure are responsible not only for the organoleptic properties, like flavors and aromas, of the broccoli sprouts, but also for their nutritional value and health properties [59].

Finally, we identified an increase in the metabolism of PUFA (Figure 4 and Tables S1–S4). Bioactive lipid mediators derived from both the enzymatic and non-enzymatic oxygenation of PUFA are known to play key roles in the life cycle of plants, including the regulation of the final maturation processes and the release of pollen (Table S1) [60]. Although our analyses were not designed for analyzing such unstable and low-abundance species, we found a significant increase in PUFA-derived hexenal species in the broccoli sprouts grown under conditions of light/dark cycle, compared with conditions of continuous dark (Table S1) [61]. Intriguingly, in addition to the chlorophyll and lipid metabolism (steroids, carotenoids, polyketides, arachidonic acid and unsaturated fatty acids metabolites), we also reported major alterations in the diterpenoid metabolism and indole alkaloid biosynthesis as consequence of light exposure in broccoli sprouts (Figure 4 and Tables S1–S4). Further, targeted measurements would be needed to establish the validity of these molecular changes.

Three of the main limitations of our metabolomic approach included: (1) the limited portion of the metabolites currently present in databases and/or identified using current experimental technology; (2) the limited availability of known metabolic networks and biochemical pathways that could allocate all of the identified metabolites; and (3) the lack of subclass differentiation for some classes of metabolites or lipids that may have independent metabolism and biological activity. Despite such limitations, using such an untargeted metabolomic approach, we were able to determine, in an unbiased fashion, a set of significant and coordinated alterations in major metabolic pathways activated by light exposure during growth in broccoli sprouts (Tables S2–S4). Further investigation is undergoing to establish the validity of these initial findings and determine their physiological roles.

To the best of our knowledge, the impact of each metabolic pathway on the molecular phenotype of broccoli sprouts after light exposure has not been reported before and this is the first comprehensive and unbiased study of its kind looking simultaneously to both lipid and polar metabolites. Furthermore, the coordinated metabolic alterations, which we were able to determine using our omics approach, allowed us to obtain a unique view of the synchronized plant metabolism during growth under light exposure. Such results could allow prioritizing future research and focusing on the predominant molecular changes occurring during light exposure.

## 3. Experimental Section

### 3.1. Growth Conditions

Broccoli seeds, (*Brassica oleracea* L. var. *botrytis* subvar. *cymosa*), purchased from SUBA&UNICO (Longiano, Italy), were germinated in the Vitaseed sprouter germination cylinder (Vitaseed AG, Switzerland) inside the phytotron and kept until harvesting in the germination cylinder. The seeds were grown hydroponically for five days at 21 °C in a plant-growth chamber (Weiss Gallenkamp, Loughborough, UK). The chamber was equipped with fluorescent tubes, PHILIPS Master TL-D 36W/840, cool-white. The tubes provided a photosynthetic photon flux density of 110 mmol·m^−2^·s^−1^. Two light regimes were adopted: (1) dark; and (2) light (16 h light/8 h dark cycle), *n* = 3 per group.

### 3.2. Sample Preparation

Sprout samples, collected from the germination cylinder, were immediately frozen in liquid nitrogen and stored at −80 °C. Metabolite extraction was conducted as previously reported [7]. Briefly, frozen sprouts were ground to a fine powder in a Waring blender, which was cooled with liquid nitrogen. Each sample of broccoli sprouts was extracted with methanol (sample-to-solvent ratio = 1:25 *w*/*v*) at 70 °C for 30 min while vortex mixing. The samples were successively centrifuged (4000 rpm, 30 min, 4 °C), the supernatants collected, and the solvent completely removed, under vacuum at 40 °C, using a rotary evaporator. The dried samples were dissolved in methanol and filtered through 0.20-μm syringe PVDF filters before MS analysis.

### 3.3. UPLC Conditions

Hydrophobic metabolites were separated using an ACQUITY UPLC system (Waters Corporation, Milford, MA, USA) equipped with a CSH C18 column (2.1 × 100 mm ID, 1.7 µm). A gradient elution was performed. Mobile phase A was composed of 60:40 (*v*/*v*) 10 mM ammonium formate in acetonitrile/water. Mobile phase B was composed of 10 mM formate in isopropanol/acetonitrile. The elution gradient was as follows: 0–2 min, 40%–43% B; 2.0–2.1 min, 43%–50% B; 2.1–12 min, 50%–54% B; 12–12.1 min, 54%–70% B; 12.1–18 min, 70%–99% B; 18–18.1 min, 99%–40% B; and 18.1–20 min, 40% B. The column was kept at 55 °C; the flow rate was 0.4 mL/min and the injection volume 5 μL.

Polar metabolites were separated using an ACQUITY UPLC system (Waters Corporation, Milford, MA, USA) fitted with a BEH HILIC column (2.1 mm × 100 mm ID, 1.7 µm). Mobile phase A was composed of 95:5 acetonitrile/water (*v*/*v*) containing 10 mM ammonium acetate (pH 8.0). Mobile phase B was composed of 50:50 acetonitrile/water (*v*/*v*) containing 10 mM ammonium acetate (pH 8.0). A 10-min linear gradient, from 100% to 80% A, with a 3-min re-equilibration time, was applied. The column was kept at 30 °C; the flow rate was 0.5 mL/min and the injection volume 5 μL.

### 3.4. MS Conditions

MS analyses were performed on an ion-mobility-enabled quadrupole, time-of-flight (QTOF) mass spectrometer (Synapt G2-S, Waters Corporation, Milford, MA, USA). Data were acquired, from 50 to 1500 *m*/*z* in both positive and negative electrospray ionization modes. The mass spectrometer was operated under the following conditions: capillary voltage 2.0 KV (+ve) and 1.0 KV (−ve); cone voltage 30 V; transfer CE ramp 20 to 50 V; source temperature 120 °C; desolvation temperature 550 °C; cone gas 50 L/h; MS gas nitrogen. Data were collected in two channels: low collision energy (6.0 V), for the molecular ions, and high collision energy (15–40 V), for product ions. The ion-mobility gas was nitrogen, and the T-wave velocity and height were 900 m/s and 40 V, respectively.

### 3.5. Data Processing and Analysis

Data processing and analysis was conducted using Progenesis QI Informatics (Nonlinear Dynamics, Newcastle, UK) [19]. Each UPLC-MS run was imported as an ion-intensity map, including *m*/*z* and retention time. These ion maps were then aligned in the retention-time direction. From the aligned runs, an aggregate run representing the compounds in all samples was used for peak picking. This aggregate was then compared with all runs, so that the same ions are detected in every run. Isotope and adduct deconvolution was applied, to reduce the number of features detected. Data were normalized according to total ion intensity. A combination of analysis of the variance (ANOVA) and multivariate statistics, including principal component analysis (PCA) and partial, least-squares discriminant analysis (PLS-DA), identified metabolites most responsible for differences between sample groups. Metabolites were identified by database searches against their accurate masses using publicly available databases, including Lipid Metabolites and Pathways Strategy (LIPID MAPS) [62], Human Metabolome database (HMDB) [63], and METLIN [64], as well as by fragmentation patterns, retention times and collision cross sections, when available. Pathway analysis, which consisted of enrichment analysis and pathway topological analysis, were conducted using Metabolomics Pathway Analysis (MetPA) within MetaboAnalyst [28]. Additional pathway over-representation and enrichment analyses with metabolite data were conducted using Metabolite Pathway Identification via coupling of global metabolite Network structure and metabolomic profile (MPINet) [29] and Integrated Molecular Pathway Level Analysis (IMPaLA) [30].

## 4. Conclusions

In this study, we developed and applied an unbiased metabolomics approach to determine the metabolic phenotypes of broccoli sprouts grown under conditions of light/dark cycle, compared with conditions of complete darkness. Our approach offered a bird’s-eye view of the metabolism of broccoli sprouts, allowing us to determine the effect of light simultaneously on a broad range of metabolites and lipids. Our study indicated the synchronized activation and coordination of specific metabolic pathways in broccoli sprouts exposed to light, which might ultimately affect their cellular physiology and nutritional value. In particular, we highlighted a predominant role for lipid metabolism in the light-induced molecular remodeling of broccoli sprouts. Exposure to light during growth affected the chlorophyll metabolism as well as major lipid biochemical pathways essential for engaging the photosynthetic machinery. These pathways include the steroid, carotenoid, and PUFA metabolism. We also observed that light exposure induced changes in the levels of polyketides, including flavonoids and oxylipins, which are related to plant growth and maturation and, potentially, their defense mechanisms against herbivores and abiotic stresses. Although our findings are not meant to be conclusive and need to be further verified, by offering a snapshot of the impact of environmental stimuli on the overall plant biochemical pathways, our untargeted metabolomics approach can provide a good starting point to generate novel hypotheses and support novel venues of investigations.

## Supplementary Materials

Supplementary materials can be found at http://www.mdpi.com/1422-0067/16/6/13678/s1.

## References

[1] Cartea M.E., Velasco P. (2008). Glucosinolates in Brassica foods: Bioavailability in food and significance for human health. Phytochem. Rev..

[2] Traka M., Mithen R. (2009). Glucosinolates, isothiocyanates and human health. Phytochem. Rev..

[3] Verkerk R., Schreiner M., Krumbein A., Ciska E., Holst B., Rowland I., de Schrijver R., Hansen M., Gerhäuser C., Mithen R. (2009). Glucosinolates in Brassica vegetables: The influence of the food supply chain on intake, bioavailability and human health. Mol. Nutr. Food Res..

[4] Armah C.N., Traka M.H., Dainty J.R., Defernez M., Janssens A., Leung W., Doleman J.F., Potter J.F., Mithen R.F. (2013). A diet rich in high-glucoraphanin broccoli interacts with genotype to reduce discordance in plasma metabolite profiles by modulating mitochondrial function. Am. J. Clin. Nutr..

[5] Pérez-Balibrea S., Moreno D.A., García-Viguera C. (2008). Influence of light on health-promoting phytochemicals of broccoli sprouts. J. Sci. Food Agric..

[6] Astarita G., Langridge J. (2013). An emerging role for metabolomics in nutrition science. J. Nutrigenet. Nutrigenomics.

[7] Maldini M., Baima S., Morelli G., Scaccini C., Natella F. (2012). A liquid chromatography-mass spectrometry approach to study “glucosinoloma” in broccoli sprouts. J. Mass Spectrom..

[8] Aires A., Rosa E., Carvalho R. (2006). Effect of nitrogen and sulfur fertilization on glucosinolates in the leaves and roots of broccoli sprouts (*Brassica oleracea* var. *italica*). J. Sci. Food Agric..

[9] Velasco P., Francisco M., Moreno D.A., Ferreres F., García-Viguera C., Cartea M.E. (2011). Phytochemical fingerprinting of vegetable Brassica oleracea and Brassica napus by simultaneous identification of glucosinolates and phenolics. Phytochem. Anal..

[10] Park W.T., Kim J.K., Park S., Lee S.-W., Li X., Kim Y.B., Uddin M.R., Park N.I., Kim S.-J., Park S.U. (2012). Metabolic profiling of glucosinolates, anthocyanins, carotenoids, and other secondary metabolites in kohlrabi (*Brassica oleracea* var. *gongylodes*). J. Agric. Food Chem..

[11] Guo R., Yuan G., Wang Q. (2011). Effect of sucrose and mannitol on the accumulation of health-promoting compounds and the activity of metabolic enzymes in broccoli sprouts. Sci. Hortic..

[12] Guzman I., Yousef G.G., Brown A.F. (2012). Simultaneous extraction and quantitation of carotenoids, chlorophylls, and tocopherols in brassica vegetables. J. Agric. Food Chem..

[13] Sun J., Xiao Z., Lin L.-Z., Lester G.E., Wang Q., Harnly J.M., Chen P. (2013). Profiling polyphenols in five *Brassica* species microgreens by UHPLC-PDA-ESI/HRMS*^n^*. J. Agric. Food Chem..

[14] Ahmadiani N., Robbins R.J., Collins T.M., Giusti M.M. (2014). Anthocyanins contents, profiles, and color characteristics of red cabbage extracts from different cultivars and maturity stages. J. Agric. Food Chem..

[15] Quanbeck S.M., Brachova L., Campbell A.A., Guan X., Perera A., He K., Rhee S.Y., Bais P., Dickerson J.A., Dixon P. (2012). Metabolomics as a hypothesis-generating functional genomics tool for the annotation of Arabidopsis thaliana genes of “unknown function”. Front. Plant Sci..

[16] Fiehn O. (2002). Metabolomics-the link between genotypes and phenotypes. Plant Mol. Biol..

[17] Martinis J., Kessler F., Glauser G. (2011). A novel method for prenylquinone profiling in plant tissues by ultra-high pressure liquid chromatography-mass spectrometry. Plant Methods.

[18] Eugeni Piller L., Besagni C., Ksas B., Rumeau D., Brehelin C., Glauser G., Kessler F., Havaux M. (2011). Chloroplast lipid droplet type II NAD(P)H quinone oxidoreductase is essential for prenylquinone metabolism and vitamin K_1_ accumulation. Proc. Natl. Acad. Sci. USA.

[19] Paglia G., Williams J.P., Menikarachchi L., Thompson J.W., Tyldesley-Worster R., Halldorsson S., Rolfsson O., Moseley A., Grant D., Langridge J. (2014). Ion mobility derived collision cross sections to support metabolomics applications. Anal. Chem..

[20] Gonzales G.B., Raes K., Coelus S., Struijs K., Smagghe G., van Camp J. (2014). Ultra (high)-pressure liquid chromatography–electrospray ionization-time-of-flight-ion mobility-high definition mass spectrometry for the rapid identification and structural characterization of flavonoid glycosides from cauliflower waste. J. Chromatogr. A.

[21] Dong W., Wang P., Meng X., Sun H., Zhang A., Wang W., Dong H., Wang X. (2012). Ultra-performance Liquid Chromatography—High-definition Mass Spectrometry analysis of constituents in the root of Radix Stemonae and those absorbed in blood after oral administration of the extract of the crude drug. Phytochem. Anal..

[22] Sun H., Ni B., Zhang A., Wang M., Dong H., Wang X. (2012). Metabolomics study on Fuzi and its processed products using ultra-performance liquid-chromatography/electrospray-ionization synapt high-definition mass spectrometry coupled with pattern recognition analysis. Analyst.

[23] Pacini T., Fu W., Gudmundsson S., Chiaravalle A.E., Brynjolfson S., Palsson B.O., Astarita G., Paglia G. (2015). Multidimensional analytical approach based on UHPLC-UV-Ion Mobility-MS for the screening of natural pigments. Anal. Chem..

[24] Paglia G., Angel P., Williams J.P., Richardson K., Olivos H.J., Thompson J.W., Menikarachchi L., Lai S., Walsh C., Moseley A. (2015). Ion mobility-derived collision cross section as an additional measure for lipid fingerprinting and identification. Anal. Chem..

[25] Sun J., Baker A., Chen P. (2011). Profiling the indole alkaloids in yohimbe bark with ultra-performance liquid chromatography coupled with ion mobility quadrupole time-of-flight mass spectrometry. Rapid Commun. Mass Spectrom..

[26] Stopka S.A., Shrestha B., Maréchal É., Falconet D., Vertes A. (2014). Metabolic transformation of microalgae due to light acclimation and genetic modifications followed by laser ablation electrospray ionization mass spectrometry with ion mobility separation. Analyst.

[27] Fu W., Magnúsdóttir M., Brynjólfson S., Palsson B.Ø., Paglia G. (2012). UPLC-UV-MSE analysis for quantification and identification of major carotenoid and chlorophyll species in algae. Anal. Bioanal. Chem..

[28] Xia J., Mandal R., Sinelnikov I.V., Broadhurst D., Wishart D.S. (2012). MetaboAnalyst 2.0—A comprehensive server for metabolomic data analysis. Nucleic Acids Res..

[29] Li F., Xu Y., Shang D., Yang H., Liu W., Han J., Sun Z., Yao Q., Zhang C., Ma J. (2014). MPINet: Metabolite pathway identification via coupling of global metabolite network structure and metabolomic profile. Biomed. Res. Int..

[30] Cavill R., Kamburov A., Ellis J.K., Athersuch T.J., Blagrove M.S., Herwig R., Ebbels T.M., Keun H.C. (2011). Consensus-phenotype integration of transcriptomic and metabolomic data implies a role for metabolism in the chemosensitivity of tumour cells. PLoS Comput. Biol..

[31] Chory J., Chatterjee M., Cook R., Elich T., Fankhauser C., Li J., Nagpal P., Neff M., Pepper A., Poole D. (1996). From seed germination to flowering, light controls plant development via the pigment phytochrome. Proc. Natl. Acad. Sci. USA.

[32] Clouse S.D., Sasse J.M. (1998). Brassinosteroids: Essential regulators of plant growth and development. Annu. Rev. Plant Biol..

[33] Phillip D., Ruban A.V., Horton P., Asato A., Young A.J. (1996). Quenching of chlorophyll fluorescence in the major light-harvesting complex of photosystem II: A systematic study of the effect of carotenoid structure. Proc. Natl. Acad. Sci. USA.

[34] Vershinin A. (1999). Biological functions of carotenoids–diversity and evolution. Biofactors.

[35] Klyachko-Gurvich G.L., Tsoglin L.N., Doucha J., Kopetskii J., Semenenko V.E. (1999). Desaturation of fatty acids as an adaptive response to shifts in light intensity 1. Physiol. Plant..

[36] Gombos Z., Wada H., Hideg E., Murata N. (1994). The unsaturation of membrane lipids stabilizes photosynthesis against heat stress. Plant Physiol..

[37] Calder P.C., Yaqoob P. (2009). Understanding omega-3 polyunsaturated fatty acids. Postgrad. Med..

[38] Simopoulos A.P. (1999). Essential fatty acids in health and chronic disease. Am. J. Clin. Nutr..

[39] Sies H., Stahl W. (2003). Non-nutritive bioactive food constituents of plants: Lycopene, lutein and zeaxanthin. Int. J. Vitam. Nutr. Res..

[40] Stahl W., Sies H. (2005). Bioactivity and protective effects of natural carotenoids. Biochim. Biophys. Acta.

[41] Jones P.J., MacDougall D.E., Ntanios F., Vanstone C.A. (1997). Dietary phytosterols as cholesterol-lowering agents in humans. Can. J. Physiol. Pharmacol..

[42] Awad A.B., Fink C.S. (2000). Phytosterols as anticancer dietary components: Evidence and mechanism of action. J. Nutr..

[43] Glueck C.J., Speirs J., Tracy T., Streicher P., Illig E., Vandegrift J. (1991). Relationships of serum plant sterols (phytosterols) and cholesterol in 595 hypercholesterolemic subjects, and familial aggregation of phytosterols, cholesterol, and premature coronary heart disease in hyperphytosterolemic probands and their first-degree relatives. Metabolism.

[44] Hollman P.C.H., Katan M. (1999). Dietary flavonoids: Intake, health effects and bioavailability. Food Chem. Toxicol..

[45] Plumb G.W., Price K.R., Modes M.J., Williamson G. (1997). Antioxidant properties of the major polyphenolic compounds in broccoli. Free Radic. Res..

[46] Gorelik S., Lapidot T., Shaham I., Granit R., Ligumsky M., Kohen R., Kanner J. (2005). Lipid peroxidation and coupled vitamin oxidation in simulated and human gastric fluid inhibited by dietary polyphenols: Health implications. J. Agric. Food Chem..

[47] Cartea M.E., Francisco M., Soengas P., Velasco P. (2011). Phenolic compounds in Brassica vegetables. Molecules.

[48] Jahangir M., Abdel-Farid I.B., Choi Y.H., Verpoorte R. (2008). Metal ion-inducing metabolite accumulation in Brassica rapa. J. Plant Physiol..

[49] Podsędek A. (2007). Natural antioxidants and antioxidant capacity of Brassica vegetables: A review. LWT-Food Sci. Technol..

[50] Fahey J.W., Zhang Y., Talalay P. (1997). Broccoli sprouts: An exceptionally rich source of inducers of enzymes that protect against chemical carcinogens. Proc. Natl. Acad. Sci. USA.

[51] Jahangir M., Kim H.K., Choi Y.H., Verpoorte R. (2009). Health-affecting compounds in *Brassicaceae*. Compr. Rev. Food Sci. Food Saf..

[52] Zhang Y. (2012). The molecular basis that unifies the metabolism, cellular uptake and chemopreventive activities of dietary isothiocyanates. Carcinogenesis.

[53] Pérez-Balibrea S., Moreno D.A., García-Viguera C. (2010). Glucosinolates in broccoli sprouts (*Brassica oleracea* var.* italica*) as conditioned by sulphate supply during germination. J. Food Sci..

[54] Moreno D.A., Carvajal M., López-Berenguer C., García-Viguera C. (2006). Chemical and biological characterisation of nutraceutical compounds of broccoli. J. Pharm. Biomed. Anal..

[55] Ciska E., Martyniak-Przybyszewska B., Kozlowska H. (2000). Content of glucosinolates in cruciferous vegetables grown at the same site for two years under different climatic conditions. J. Agric. Food Chem..

[56] Pérez-Balibrea S., Moreno D.A., García-Viguera C. (2011). Genotypic effects on the phytochemical quality of seeds and sprouts from commercial broccoli cultivars. Food Chem..

[57] Vallejo F., Tomás-Barberán F., García-Viguera C. (2002). Glucosinolates and vitamin C content in edible parts of broccoli florets after domestic cooking. Eur. Food Res. Technol..

[58] Goodspeed D., Liu J.D., Chehab E.W., Sheng Z., Francisco M., Kliebenstein D.J., Braam J. (2013). Postharvest circadian entrainment enhances crop pest resistance and phytochemical cycling. Curr. Biol..

[59] Talalay P., Fahey J.W. (2001). Phytochemicals from cruciferous plants protect against cancer by modulating carcinogen metabolism. J. Nutr..

[60] McConn M. (1996). The critical requirement for linolenic acid is pollen development, not photosynthesis, in an Arabidopsis mutant. Plant Cell Online.

[61] Berdyshev E.V. (2011). Mass spectrometry of fatty aldehydes. Biochim. Biophys. Acta.

[62] Fahy E., Subramaniam S., Murphy R.C., Nishijima M., Raetz C.R., Shimizu T., Spener F., van Meer G., Wakelam M.J., Dennis E.A. (2009). Update of the LIPID MAPS comprehensive classification system for lipids. J. Lipid Res..

[63] Wishart D.S., Jewison T., Guo A.C., Wilson M., Knox C., Liu Y., Djoumbou Y., Mandal R., Aziat F., Dong E. (2013). HMDB 3.0—The Human Metabolome Database in 2013. Nucleic Acids Res..

[64] Smith C.A., O’Maille G., Want E.J., Qin C., Trauger S.A., Brandon T.R., Custodio D.E., Abagyan R., Siuzdak G. (2005). METLIN: A metabolite mass spectral database. Ther. Drug Monit..

