# Supplementary material for: Untargeted Metabolomics Reveals Predominant Alterations in Lipid Metabolism Following Light Exposure in Broccoli Sprouts
Source: Int J Mol Sci. 2015 Jun 15;16(6):13678–91. doi: 10.3390/ijms160613678 (PMC4490517; doi:10.3390/ijms160613678)
Supplement: Supplementary file 1 [file ijms-16-13678-s001.zip › ijms-80155-Supplementary Information/ijms-80155-Figure S1.pdf]

# Supplementary Information

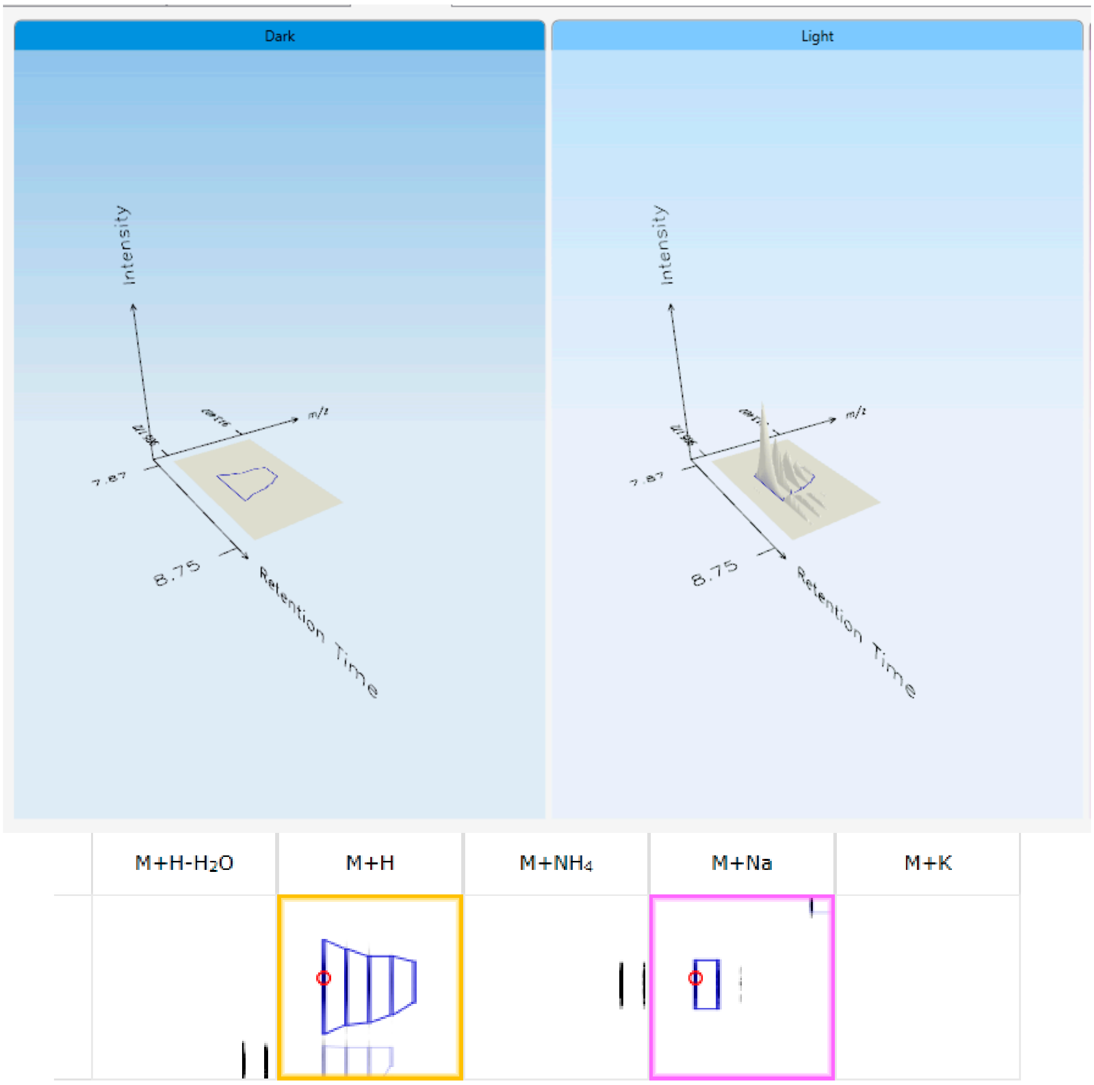


(A)

**METLIN**

**Metabolites**

Mass 907.5210 with 5 ppm mass accuracy

Change Query

**Total: 1 Metabolite**

| METLIN ID | MASS | Δppm | NAME | MS/MS | STRUCTURE |
|---|---|---|---|---|---|
| 3704 | [M+H]+ **m/z** **907.5219** M 906.5146 | 0 | **Chlorophyll b** *Formula:* C55H70MgN4O6 *CAS:* 519-62-0 | View | 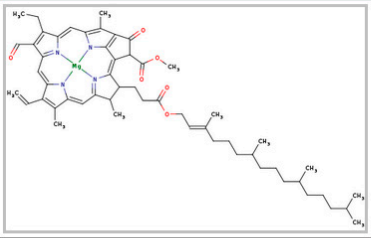 |

**Figure S1.** *Cont.*

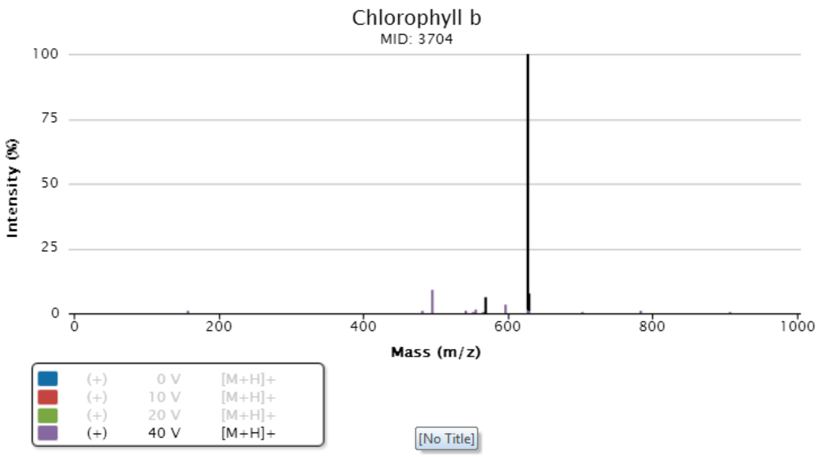


(**B**)

**Figure S1.** The statistical identification of metabolic alterations was followed by a review of the measurements using 3D montage and adducts deconvolution (**A**) and a search on local or online databases (e.g., METLIN, (**B**)) for structural identification. In this example, a database search lead to a putative structure of a chlorophyll *b*, which was detected only in broccoli sprouts grown in light conditions (**A**).
